# Artificial intelligence in lung cancer screening: Detection, classification, prediction, and prognosis

**DOI:** 10.1002/cam4.7140

**Published:** 2024-04-05

**Authors:** Wu Quanyang, Huang Yao, Wang Sicong, Qi Linlin, Zhang Zewei, Hou Donghui, Li Hongjia, Zhao Shijun

**Affiliations:** ^1^ Department of Diagnostic Radiology National Cancer Center/National Clinical Research Center for Cancer/Cancer Hospital, Chinese Academy of Medical Sciences and Peking Union Medical College Beijing China; ^2^ Magnetic Resonance Imaging Research General Electric Healthcare (China) Beijing China; ^3^ PET‐CT Center National Cancer Center/National Clinical Research Center for Cancer/Cancer Hospital, Chinese Academy of Medical Sciences and Peking Union Medical College Beijing China

**Keywords:** artificial intelligence, computed tomography, convolutional neural network, deep learning, lung cancer

## Abstract

**Background:**

The exceptional capabilities of artificial intelligence (AI) in extracting image information and processing complex models have led to its recognition across various medical fields. With the continuous evolution of AI technologies based on deep learning, particularly the advent of convolutional neural networks (CNNs), AI presents an expanded horizon of applications in lung cancer screening, including lung segmentation, nodule detection, false‐positive reduction, nodule classification, and prognosis.

**Methodology:**

This review initially analyzes the current status of AI technologies. It then explores the applications of AI in lung cancer screening, including lung segmentation, nodule detection, and classification, and assesses the potential of AI in enhancing the sensitivity of nodule detection and reducing false‐positive rates. Finally, it addresses the challenges and future directions of AI in lung cancer screening.

**Results:**

AI holds substantial prospects in lung cancer screening. It demonstrates significant potential in improving nodule detection sensitivity, reducing false‐positive rates, and classifying nodules, while also showing value in predicting nodule growth and pathological/genetic typing.

**Conclusions:**

AI offers a promising supportive approach to lung cancer screening, presenting considerable potential in enhancing nodule detection sensitivity, reducing false‐positive rates, and classifying nodules. However, the universality and interpretability of AI results need further enhancement. Future research should focus on the large‐scale validation of new deep learning‐based algorithms and multi‐center studies to improve the efficacy of AI in lung cancer screening.

## 1 INTRODUCTION

Lung cancer (LC) is the primary cause of cancer‐related mortality worldwide and the second most frequently diagnosed cancer globally, as reported by GLOBOCAN 2020.^1^ Patients with distant‐stage lung cancer exhibit a 5‐year relative survival rate of 6%, whereas those diagnosed at a regional stage show a rate of 33%.^2^ Due to the lack of early symptoms, patients often miss the optimal treatment window, making early screening crucial for the prevention and management of lung cancer.^3^, ^4^ A non‐randomized controlled trial conducted by the International Early Lung Cancer Action Program (I‐ELCAP) reported that more than 80% of LC cases can be discovered in their earliest stages using low‐dose computed tomography (LDCT) screening. The 10‐year relative survival rate is up to 88% if treatment is administered quickly enough.^5^ According to the National Lung Screening Trial (NLST) in the US and the Dutch–Belgian Randomized Lung Cancer Screening Trial (NELSON) in Europe, screening with LDCT reduces LC mortality.^6^, ^7^ Currently, LDCT is the only internationally recognized screening method that has demonstrated a decrease in mortality rates in high‐risk populations for LC.^8^ The NLST found that 26.8% of participants had lung nodules larger than 4 mm.^9^ Pulmonary nodules are clinically relevant as they can be the initial manifestation of LC. In general, pulmonary nodules refer to spherical lung opacities or irregular lung lesions that are sized from 3 to 30 mm and can appear as a single entity or in multiples. Pulmonary nodules display diverse characteristics including quantity (single or multiple), size, shape (regular or irregular), margins (smooth, lobulated, or spiculated), location (well‐defined, near the pleura, or near blood vessels), and density (solid, part‐solid, or non‐solid). There may be a correlation between several nodule characteristics and a higher likelihood of LC, including nodule diameter, position of the superior lobe, and solid components.^10^, ^11^ The nodule volume and mass can reveal information about natural evolutionary development.^12^, ^13^ Therefore, it is especially important for radiologists to accurately detect nodules and correctly identify their characteristics. However, many nodules are in close proximity to the pleura or blood vessels and may be easily missed. In numerous instances, distinguishing the contour of a nodule is difficult because of inflammation or pleural effusion. To summarize, the variety and unpredictability of pulmonary nodules significantly complicate their accurate detection and diagnosis.

**FIGURE 1:**
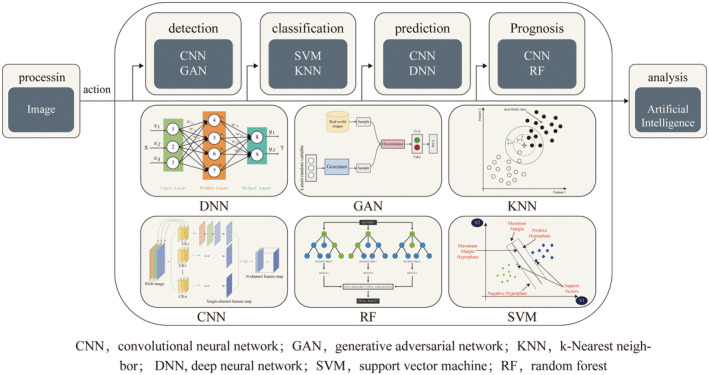
Function diagram of the use of AI for detection, classification, prediction, and prognosis of lung cancer screening. AI, artificial intelligence.

With advances in computer technology, artificial intelligence (AI) has rapidly emerged and is applied in various medical settings (Figure 1). AI is a field in computer science that uses available data to predict or categorize objects. It encompasses key elements such as training datasets, preprocessing techniques, algorithms for creating predictive models, and pre‐trained models for accelerating model development and leveraging previous experience.^14^ The growth in the application of AI to radiology is founded upon two key pillars. The first pillar is the expansion of machine learning (ML). ML employs statistical methods to automatically construct rules for its algorithms using existing training data. Thus, the primary objective of ML is to quickly and efficiently recognize patterns within large datasets. It can produce results that are more accurate than manual human evaluations;^15^ There are three distinct forms of ML: supervised learning, unsupervised learning, and reinforcement learning.^16^, ^17^ The learner parameter is changed during supervised learning to get closer to the desired outcome. In other words, the correct answer label is learned from the training data and a learning algorithm is constructed whose output is the correct answer label. Next, models are evaluated to see if they produce results that are reasonably close to the “right label” when applied to sets of unknown data. In the area of image recognition, this ML technique is most frequently employed for classification and regression tasks.^18^ Supervised learning necessitates a large amount of training data, including labeled data, which can be challenging to acquire in the medical and biological fields. Conversely, unsupervised learning is another type of ML that utilizes only input data, without any accompanying “correct answer” data to guide the learning process. Reinforcement learning, the final type of ML, updates the learning model through a trial‐and‐error approach to determine the optimal course of action for a given situation. Frequently utilized ML algorithms comprise support vector machines (SVMs), decision trees (DTs), and Bayesian networks (BNs).

**FIGURE 2:**
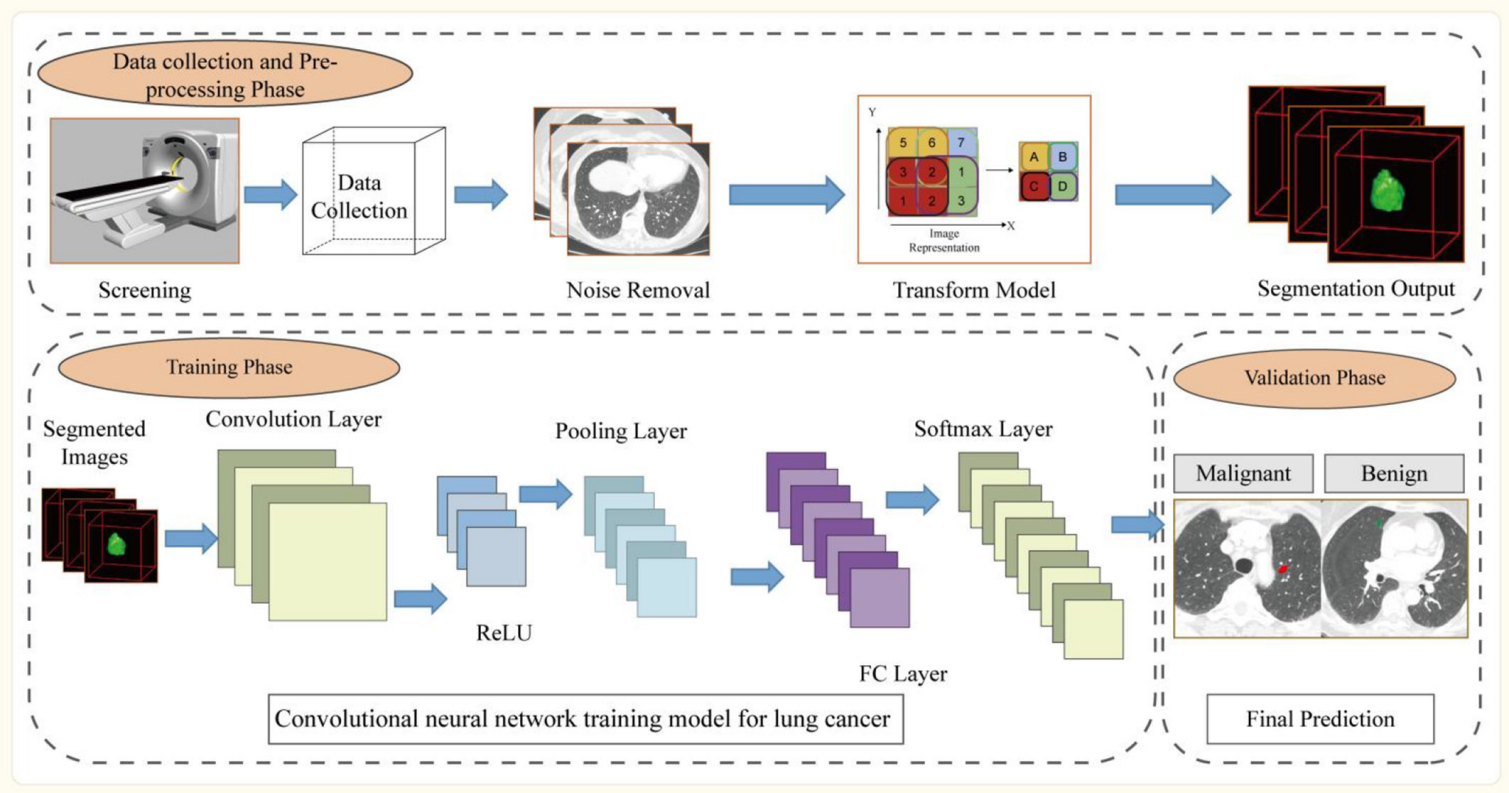
Convolutional neural network training model for lung cancer.

The second pillar is represented by the expansion of the AI branch, known as deep learning (DL). In contrast to traditional ML systems that depend on human‐engineered feature extraction and data structuring from images, DL algorithms use raw data and are capable of learning the necessary representations for pattern recognition independently.^19^ DL, a type of representational learning, enables the creation of sophisticated multi‐layer neural network structures that automatically uncover new knowledge through the analysis of input data at multiple levels.^20^ The simultaneous feature selection and model fitting technique is an efficient method for constructing models using automated procedures and high‐volume data.^14^, ^21^ DL systems have the ability to convert input images into valuable outputs, including object detection through localization, image segmentation through pixel labeling, and image classification into various categories.^22^ The convolutional neural network (CNN) is the most widely used architecture for analysis of medical images through DL. CNN encompasses a diverse and rich set of algorithms (Data S1), which are meticulously designed to meet its specific purposes and application requirements. CNNs are designed with multiple sequential layers of convolution, where the representation generated by each layer (beginning with the raw input data) is passed on to the subsequent layer, transforming into increasingly abstract representations.^19^, ^23^ As the computational capacity of computers increases, particularly graphics processing units, DL has established itself as the preferred approach for analyzing medical images, showing impressive results in oncology applications ranging from tumor identification to prognosis prediction (Figure 2). This article aimed to explore the use of AI in CT screening, including lung segmentation, nodule detection, nodule classification, nodule subtype prediction, and prognosis.

## 2 LUNG SEGMENTATION

Before performing lung nodule detection, it is necessary to segment the lung. The purpose of lung lobe segmentation is to accurately define the anatomical structure of lung lobes, enabling the differentiation of regions associated with lung nodules. Many algorithms have been created specifically for this task. The main conventional approaches include thresholding,^24^ region growing algorithm,^25^, ^26^, ^27^ morphological filters,^28^, ^29^ connected component analysis,^30^, ^31^ and the boundary tracking algorithm.^32^, ^33^ A number of improved techniques based on traditional methods have further improved the efficacy of lung segmentation and optimized the shortcomings of traditional methods. Shi et al. focused on two‐dimensional (2D) region‐growing algorithms. An optimized threshold was applied to transform the smoothed slice into a binary image by utilizing an algorithm that is founded on seed‐based random walks, allowing for the segregation of lung regions from thorax regions.^34^ Soliman et al. proposed a learnable multi‐graph random field (MGRF) system that integrates independent submodels for visual appearance and adaptive lung shape. The Dice index was 98.5%, and the average overlap between the learnable model and expert segmentation was 98.0%.^35^ Filho et al. proposed a 3D adaptive crisp active contour method (3D ACACM) framework. This framework is initiated with a sphere placed within the lung, which is shaped by forces toward the lung borders to be segmented. The process is executed iteratively with the aim of minimizing the energy function related to the 3D deformable model, enabling the segmentation of both normal and pathological lungs.^36^ Zhang and Fischer successfully implemented advanced techniques in their statistical shape model and AI‐RAD companion framework. These methods encompass statistical finite element analysis and enhancement of 3D lung segmentation through adversarial neural network training.^37^, ^38^

Recently, DL algorithms, particularly CNNs, have shown promising results in automatically segmenting lungs from CT images. Several advanced CNNs are currently available for lung segmentation, including 3D U‐Net,^39^ DenseV‐Net,^40^ RPLS‐Net,^41^ and nnU‐Net.^42^ Using 3D U‐Net, Park et al. devised a fully automated method for lung‐lobe segmentation that was rigorously validated using both an internal and external dataset and it exhibited a reasonable level of segmentation accuracy and computational efficiency. Additionally, this method could be adapted and utilized in clinical settings to address lung lobe segmentation in severe lung diseases.^39^ Peng et al. proposed an algorithm based on DL called nnU‐Net, which is capable of auto‐configuring itself, including preprocessing, network architecture, training, and post‐processing. The pre‐operative nnU‐Net model achieved a dice similarity coefficient (DSC) of 0.964, and the model had a DSC of 97.3% after lobectomy.^42^ The effective methods that have been proposed and selected are summarized in Table 1.

**TABLE 1:** Recent artificial intelligence‐based approaches for lung lobe segmentation.

Year	Authors	Method	No. of cases	Quality index	Quality index value
2016	Shi et al.^34^	Thresholding	23	Overlap measure	98.40%
2017	Soliman et al.^35^	Shape‐based	105	Dice index	98.50%
2017	Rebouças Filho et al.^36^	Deformable model	40	F‐measure	99.22%
2019	Zhang et al.^37^	Statistical finite element analysis	20	N/A	N/A
2020	Fischer et al.^38^	AI‐RAD	137	N/A	N/A
2020	Park et al.^39^	3D U‐Net	196	Dice index	97.00%
				Jaccard index	94.00%
2020	Dong et al.^99^	MV‐SIR	874	Dice index	92.60%
2021	Liu et al.^41^	RPLS‐Net	32	Dice index	94.21%
2022	Pang et al.^42^	nnU‐Net	865	Dice index	96.40%

## 3 NODULE DETECTION

Nodule detection consists of two main components: Candidate nodule detection and false‐positive reduction. Traditional techniques primarily encompass classical image processing techniques, comprising intensity‐based techniques (such as thresholding and region growing) and shape‐based techniques (such as the 3D detection box, spherical shape enhancement filter, and graph‐cut method). Feature engineering algorithms were commonly applied for nodule detection before the advent of DL.^43^, ^44^, ^45^ The features of tumors, such as intensity, texture, and morphology, were precisely extracted from CT data through manual processes and then utilized as inputs for various ML classifiers, including SVMs and random forest (RF). In contrast, AI methods, especially CNN‐based methods, are capable of adapting and developing appropriate representations through a fully data‐driven approach without relying on manually derived lung nodule attributes. They boast a high level of automation and minimize the need for manual intervention.^46^, ^47^

### 3.1 Candidate nodule detection

The increasing popularity of DL has led to the proposal of many effective algorithms for nodule detection based on CNN techniques.^48^ The promising performance of CNN in pulmonary nodule segmentation tasks can be attributed to the network's capacity to learn novel features at different levels of the hierarchy. In particular, the network hierarchy architecture can capture the 2D and 3D aspects of lung nodules, which have not been previously addressed. Network architectures that are effective for nodule detection include U‐Net, region proposal networks (RPNs), residual networks (ResNets), and retinal nets. Most detection techniques can be viewed as variant versions of these network architectures.^49^, ^50^, ^51^, ^52^, ^53^, ^54^ The other type is a hybrid network consisting of multiple structures arranged in a cascade fashion.^55^, ^56^, ^57^

A groundbreaking study highlighted the ability of AI algorithms to support radiologists in diagnosing pulmonary nodules during LC screening. This study utilized a specific DL system with a multistream convolutional network architecture for categorizing lung nodules. Categorization was based on the Lung‐RADS assessment and PanCan malignancy criteria, which were deemed relevant for patient care. Compared to patch categorization using ML, this model performed better and had inter‐observer variability that was on par with that of four human radiologists.^58^ Cai et al. utilized a feature pyramid network (FPN) to extract feature maps from the input data, which was then fed into a Mask R‐CNN based on the ResNet50 architecture. Next, prospective nodule bounding boxes were created using an RPN fed with the feature maps. The proposed technique demonstrated a high sensitivity of 88.70% on the LUNA16 dataset, with an average of eight false positives per scan, thereby demonstrating its potential effectiveness.^59^ A manifold regularized classification deep neural network (MRC‐DNN), developed by Ren et al., generated a reconstructed image of an input nodule using an encoder‐decoder structure for manifold learning. During this process, a nodule manifests itself in many ways. A manifold can be classified directly using a fully connected neural network. In addition, several fusion networks have been meticulously investigated using multi‐stream topologies to seamlessly combine the strengths of multiple networks and enhance the overall performance.^60^ Nasrullah et al. employed a cutting‐edge approach for nodule detection, utilizing a combination of a Faster R‐CNN with a U‐net‐like architecture and a specially designed mixed link network (CMixNet). The volumetric CT image was divided into 96 × 96 × 96 voxel subvolumes, which were processed independently and combined to form the final nodule‐detection algorithm. This method achieved a remarkable sensitivity of 94.21% on the LIDC dataset with an average of eight false positives per scan.^61^ Yuan et al. devised a sophisticated multi‐modal fusion multi‐branch classification network to detect and categorize pulmonary nodules with high accuracy. The network incorporated a 3D ECA‐ResNet that dynamically adapted the extracted features. Feature maps from various multilayer receptive fields are integrated to obtain comprehensive multiscale unstructured characteristics. The nodules were then classified as benign or malignant based on the results of a fusion of structured and unstructured data, leveraging the strengths of multiple modalities.^62^ The effective methods proposed are selected and summarized in Table 2.

**TABLE 2:** Recent artificial intelligence‐based approaches for pulmonary nodule detection.

First author/year	Algorithm	Source of data	No. of cases	Type of validation	Main finding	Quality index value
Ciompi et al. 2017^58^	CNN: Support vector machines	Data from the MILD trial and DLCST trial	943 patients (1352 nodules) from the MILD trial	468 patients (639 nodules) from the DLCST trial	The model outperformed classical patch classification approaches classifying lung nodules and its performance was comparable to that of experienced radiologists	Average performance between the computer and observers, with an average accuracy of 72.9% versus 69.6%
				Accuracy: Compared with experienced radiologists (assessing 162 nodules from the test set)		
Cai et al. 2020^59^	Mask R‐CNN with ResNet50 architecture	Data from LUNA16 dataset	888 patients from the LUNA16 dataset	800 patients from an independent dataset from the Ali TianChi challenge	Using mask R‐CNN and the ray‐casting volume rendering algorithm can assist radiologists in diagnosing pulmonary nodules more accurately.	Mask R‐CNN of weighted loss reaches sensitivities of 88.1% and 88.7% at 1 and 4 false positives per scan
Nasrullah et al. 2019^61^	Faster R‐CNN/ CMixNet/U‐net	Data from LUNA16 dataset	888 patients from the LUNA16 dataset	1018 patients from the LIDC‐IDRI dataset	Automated lung nodule detection and classification using deep learning combined with multiple strategies aids in the reduction of misdiagnosis and false positive results in the early‐stage	The model was evaluated on LIDC‐IDRI datasets in the form of sensitivity (94%) and specificity (91%)
Ren et al. 2020^60^	MRC‐DNN	Date from LIDC‐IDRI dataset	883 patients from the LIDC‐IDRI dataset	98 patients from the LIDC‐IDRI dataset	MRC‐DNN facilitates an accurate manifold learning approach for lung nodule classification based on 3D CT images	The classification accuracy on testing data is 0.90 with sensitivity of 0.81 and specificity of 0.95
Cui et al. 2020^100^	ResNet	Lung cancer screening data from three hospitals in China	39,014 chest LDCT screening cases	Validation set (600 cases). External validation: the LUNA public database (888 studies)	The DL model was highly consistent with the expert radiologists in terms of lung nodule identification	The AUC achieved 0.90 in the LUNA dataset
Shen et al. 2019^101^	HSCNN	Date from the LIDC‐IDRI dataset	897 cases from LIDC‐IDRI dataset	Date from the LIDC‐IDRI dataset	The HSCNN model not only produces interpretable lung cancer predictions but also achieves significantly better results compared to using a 3D CNN alone	The HSCNN model achieved a mean AUC 0.856, mean accuracy of 0.842, mean sensitivity of 0.705 and mean specificity of 0.889
Yu et al. 2021^102^	3D Res U‐Net	LIDC‐IDRI	1074 CT subcases from LIDC‐IDRI	174 CT data from 1074 044 CT subcases	3D Res U‐Net can identify small nodules more effectively and improve its segmentation accuracy for large nodules	The accuracy of 3D ResNet50 is 87.3% and the AUC is 0.907
Liu et al. 2022^103^	Res‐trans	LIDC‐IDRI	848 nodules (442 benign)	Tenfold cross‐validation and compared with recently leading methods	Res‐trans networks capture local/global features can help radiologists to accurately analyze lung nodules	AUC = 0.9628 and accuracy = 0.9292
Yuan et al. 2023^62^	3D ECA‐ResNet	LUNA16/LIDC‐IDRI	1080 scans/888 scans	Comparison with state‐of‐the‐art‐methods	Multi‐modal feature fusion of structured data and unstructured data is performed to classify nodules	Accuracy (94.89%), sensitivity (94.91%), and F1‐score (94.65%) and lowest false positive rate (5.55%).
Khan et al. 2022^104^	AdaBoost‐SNMV‐CNN	LIDC‐IDRI	14,220 images (7110 2D images, 7110 3D images)	External validation: ELCAP dataset	The multiviews confer the model's good generalization and learning ability for diverse features of lung nodules	The accuracy for detecting lung nodules was 99%; sensitivity was 100% and specificity was 98%
Liu et al. 2023^105^	PiaNet	LIDC‐IDRI	302 CT scans from LIDC‐IDRI	52 CT scans from LIDC‐IDRI	Pi‐aNet is capable of more accurately detecting GGO nodules with diverse characteristics.	A sensitivity of 93.6% with only one false positive per scan
Siddiqui et al. 2023^106^	3D MLF‐DCNN	LUNA 16/LIDC‐IDRI/TCIA	27,816 CT images	5564 CT images from LUNA 16/LIDC‐IDRI/TCIA	(128 × 128 × 20)‐dimensional dataset achieved the best performance in parameters	The accuracy, sensitivity, and specificity are all 99.20%, and its specificity is 99.17%

Abbreviations: 3D, three‐dimensional; AUC, area under the curve; CNN, convolutional neural network; CT, computed tomography; ELCAP, Early Lung Cancer Action Program; MRC‐DNN, manifold regularized classification deep neural network.

### 3.2 False positive reduction

Reducing the number of false positives following the candidate nodule detection stage is of utmost importance to enhance the overall accuracy of nodule detection. According to a recent review by Schreuder et al., algorithms have lower or similar sensitivities to assessments by radiologists, but at the cost of higher false‐positive rates.^63^ In essence, false‐positive reduction can be considered a preparatory phase for nodule classification. The steps involved in reducing false positives generally include feature extraction, feature selection, and nodule classification (except for deep‐learning techniques based on CNN, which can automatically learn discriminative features). The primary objective of feature extraction is the extraction of 2D or 3D features of lung nodules and the subsequent analysis of candidate nodule images based on properties such as intensity, morphology, and texture. Nodule classification relies heavily on precise and pertinent criteria. These extracted features are then utilized by various ML classifiers, such as SVM, RF, k‐nearest neighbor classifiers, linear discriminant classifiers, and boosting classifiers, to differentiate between true nodules and non‐nodules.^64^, ^65^, ^66^ Tartar used principal component analysis to extract features and combine morphological and statistical features into a mixture of parameters, and fed the extracted parameters into various classifiers, including RF, Bagging, and Adaboost to reduce false positives.^67^ Gong presented a novel approach for the automatic detection of lung nodules by combining a 3D tensor filtering technique with local image feature analysis. This approach uses a 3D level‐set segmentation method to define the borders of potential nodule candidates precisely. The correlation feature selection subset evaluator was employed to extract the best features from the identified candidates. The final step involves training an RF classifier to categorize the candidates, resulting in improved sensitivity for detecting large nodules.^29^

Recently, several CNN‐based methods have been proposed for false positive reduction. Due to the differences in the structures of the networks, they can be divided into two categories: Advanced off‐the‐shelf CNNs and multistream heterogeneous CNNs. Kim et al. proposed a groundbreaking multiscale gradual integration CNN that significantly reduced false positives in the detection of pulmonary nodules, achieving competitive performance metrics (CPM) of 0.908 and 0.942 in two subsets of LUNA16. The advantage of this model is that it can use 3D multiscale inputs and progressively extract features from the multiscale inputs of different layers. In addition, to more effectively utilize complementary information, they employed multi‐stream feature integration to seamlessly integrate abstract‐level feature representations.^68^ Zuo et al. suggested using an embedded multi‐branch 3D CNN to detect lung nodules with lower false positives. Each branch processed a feature map from a distinct layer. All these branches are cascaded at their endpoints. Hence, characteristics from various depth layers are pooled to forecast the candidate categories. In the validation set, the accuracy and specificity were 0.978 and 0.877, respectively, with a CPM of 0.83.^69^ Masood created an innovative automated clinical decision support system for lung detection that leverages a 3D CNN architecture. The system utilizes a novel median intensity projection and introduces an innovative multiregion proposal network for the automatic selection of potential regions‐of‐interest. To minimize the false‐positive results, a computer‐aided decision (CAD) support system was adapted for integration with cloud computing. The system obtained an impressive 98.7% sensitivity at 1.97 false positives per scan.^70^ Yuan et al. recently proposed an MP‐3D‐CNN model to efficiently extract spatial information of potential nodule properties via a hierarchical structure. By adopting and concatenating three routes representing three receptive field widths into the network model, the feature information was fully retrieved and fused to dynamically adapt to the differences in shape, size, and context across the pulmonary nodules. Sensitivities of 0.952 and 0.962 were achieved at 4 and 8 false positives per scan, respectively, demonstrating exceptional performance.^71^ The effective methods proposed are summarized in Table 3.

**TABLE 3:** The latest artificial intelligence‐based methods for reducing the false positive rate.

Year	Authors	Method/identified features	Dataset	Quality index	Quality index value
2013	Tartar et al.^67^	Shape features	Dataset from Cerrahpasa Medicine Faculty, Istanbul University	Sensitivity	0.896
				Specificity	0.875
2014	Teramoto et al.^107^	Shape features, intensity	Cancer‐screening program at the East Nagoya Imaging Diagnosis Center	Sensitivity	0.83
2018	Gong et al.^29^	Intensity, shape, texture features	LUNA16/ANODE09	Sensitivity	0.8462
2019	Zuo et al.^108^	Multi‐resolution features integrated 2D CNN	LUNA16	Accuracy	0.9733
2019	Zhou et al.^95^	2/3D Models Genesis with encoder‐decoder architecture	LUNA16	AUC	0.982
2019	Kim et al.^68^	Multi‐scale gradual integration CNN	LUNA16	CPM	0.942
2020	Sun et al.^109^	S‐transform	Dataset from Sichuan Provincial People's Hospital	Sensitivity	0.9787
2020	Zuo et al.^69^	Multi‐branch 3D CNN	LUNA16	CPM	0.83
2020	Masood et al.^70^	Multi‐PRN inspired by VGG‐16	LUNA16/LIDC‐IDRI	Sensitivity	0.974
2021	Majidpourkhoei et al.^110^	CADe/CADx	LIDC‐IDRI	Accuracy	0.901
				Sensitivity	0.841
				Specificity	0.917
2021	Yuan et al.^71^	MP‐3D‐CNN	LUNA16	CPM	0.881
				Sensitivity	0.962
2023	Mkindu et al.^111^	3D residual CNN with 3D ECA	LUNA16	CPM	0.911
				Sensitivity	0.9865

Abbreviations: AUC, area under the curve; CNN, convolutional neural network; CPM, competitive performance metrics.

## 4 NODULE CLASSIFICATION

The classification of pulmonary nodules is a central aspect of LC screening. While most AI systems focus on predicting malignancy and determining the nature of a nodule, only some have been designed specifically to categorize nodule types. For instance, Savitha proposed a fully automated CAD system for the identification and classification of nodule types during LC screening. The system utilizes gray‐level covariance matrix and principal component analysis algorithms to extract feature vectors. Nodule localization was performed using SVM, Fuzzy C‐means, and RF classification algorithms. The identified nodules were then categorized into solid and sub‐solid types by extracting histogram of gradient features.^72^

The performance of the classifier is crucial for the classification of benign and malignant nodules. To better arrange the presentation of relevant papers, we split the classifiers into two groups: Conventional and DL classifiers. Although traditional ML classifiers such as SVM and RF often produce satisfactory results, they have several limitations. For example, deploying an SVM becomes challenging when dealing with multi‐classification problems and large training datasets, and typical ML classifiers require human feature extraction to obtain optimal performance. Manual feature extraction can be a labor‐intensive and intricate process, particularly in the context of medical image analysis, where diagnostic complexity and limited prior knowledge exacerbate the challenge. Indeed, despite clinicians' experience, there is a lack of understanding of the quantitative imaging features that best predict outcomes. Moreover, the manual feature extraction of lung nodule characteristics is difficult. DL algorithms possess a high degree of automation and require minimal manual intervention because they can automatically develop a relevant representation through data‐driven learning without relying on manually obtained information about the lung nodules. In addition, the knowledge acquired by DL algorithms from other domains can be transferred more easily to the domain of LC diagnosis than the knowledge gained by traditional ML algorithms.^73^, ^74^ Consequently, DL algorithms provide several benefits when assessing the LC data.

DL based on CNN has produced a variety of classification techniques:

1. Advanced off‐the‐shelf CNNs.^51^, ^75^, ^76^ To distinguish malignant from benign forms, Filho et al. used standardized taxic weights and index basic taxic weights.^77^ Topology‐based phylogenetic diversity indices were proposed for feature selection, and feature data were fed to 2D CNNs. The proposed approach demonstrated exceptional performance in the diagnosis of cancer and benignity; the obtained results showing that the accuracy, sensitivity, specificity, and area under the curve (AUC) were 92.63%, 90.7%, 93.4%, and 0.93, respectively. Xie et al.utilized a multi‐view knowledge‐based collaborative deep model to distinguish between benign and malignant lung nodules. The 3D nodule was divided into nine fixed views, each of which served as a KBC submodel. To enhance the characterization of the nodules' overall appearance, voxels, and form heterogeneity, three types of picture patches were designed for each submodel and used to fine‐tune the three pre‐trained ResNet‐50 networks. The nine submodels were integrated using an adaptive weighting approach derived from error backpropagation, and a penalty loss function was employed to reduce the false negative rate with minimal impact on the results. This approach achieved an accuracy of 91.60% and AUC of 95.70%.^78^
2. CNNs integrated with ML classifiers. Zhu et al. introduced a fully automated LC diagnostic system called DeepLung. This system featured a 3D Faster R‐CNN incorporating 3D dual‐path blocks and a U‐net‐inspired encoder‐decoder structure for nodule detection. In addition, the system employed a gradient boosting machine (GBM) equipped with 3D dual‐path network characteristics for nodule classification. The nodule classification subnetwork was validated using a public dataset from LIDC‐IDRI.^50^ Nasrullah and Zhu shared a similar research idea, but Nasrullah used the hybrid network CMixNet through R‐CNN for learning nodule features. Nasrullah's 3D‐CMixNet architecture includes a GBM for nodule classification using learned characteristics. To further reduce misdiagnosis, physiological symptoms and clinical biomarkers are combined. With the LIDC‐IDRI dataset, the proposed system was assessed based on sensitivity (94%) and specificity (91%).^61^
3. Multistream HCNNs. Liu et al. presented MTMR‐Net, a multi‐task deep model with a margin ranking loss for automated lung nodule analysis. This multi‐task deep model investigated the causal relationship between lung nodule categorization and attribute score regression. The model also incorporates a Siamese network with margin ranking loss to enhance its ability to distinguish challenging nodule scenarios. The effectiveness of the MTMR‐Net model was validated in an LIDC‐IDRI dataset.^79^ Bonavita et al. developed a malignancy classifier based on a 3D CNN, utilizing annotations from radiologists on lung nodules. This classifier was integrated into the LC classification pipeline, and its performance was compared with that of the baseline pipeline. The contribution of nodule malignancy classifiers was quantified in the prediction of LC, and the results demonstrated that the integration of these predictive models enhanced the accuracy of LC prediction.^80^
4. CNNs were trained using transfer learning algorithms. Transfer learning involves utilizing the understanding acquired by training a model on a certain task and applying it to solve new or related problems, thereby reducing the need for extensive training data. When analyzing natural images, deep CNNs have exhibited remarkable performance. However, the ability to achieve such high performance is highly dependent on a substantial number of datasets. Medical images are far from adequate in number compared to natural images, which limits the development of CNN to some extent. Therefore, transfer learning can potentially serve as an alternative approach for analyzing lung nodules in medical images through the utilization of deep CNN models. Harsono et al. developed I3DR‐Net, a one‐stage detector for detecting and classifying lung nodules that combines an FPN with a pretrained inflated 3D ConvNet (I3D) on a multiscale 3D thoracic CT scan dataset. The I3DR‐Net outperformed Retina U‐Net and U‐FRCNN, achieving a 7.9% and 7.2% increase in mean average precision (mAP) for the detection and classification of malignant nodules.^81^ The effective methods proposed are selected and summarized in Table 4.

**TABLE 4:** The latest artificial intelligence‐based methods for classifying benign and malignant nodules.

Year	Authors	Data source	Method	Quality index	Quality index value
2016	Petousis et al.^112^	NLST dataset	DBNs Including three expert‐driven DBNs and two DBNs derived from structure learning methods	AUC	>0.75
2018	Filho et al.^77^	LIDC‐IDRI	Topology‐based phylogenetic diversity indices are proposed for features engineering and selection. Feature data are fed to 2D CNNs	Accuracy	0.9263
				AUC	0.934
2018	Causey et al.^90^	LIDC‐IDRI	Training 3D CNN models and collecting output features. A 3D CNN is then used for malignancy classification based on quantitative image features	AUC	0.99
2018	Dey et al.^91^	LIDC‐IDRI	Performance comparison between 3D DCNN and 3D DenseNet variants	Accuracy	0.899
				AUC	0.9459
2019	Balagurunathan et al.^113^	NLST dataset	Optimal linear classifiers	AUC	0.85
2019	Al‐Shabi et al.^114^	LIDC‐IDRI	Deep Local‐G lobal networks containing residual blocks and non‐local blocks	AUC	0.9562
2019	Chen et al.^115^	LIDC‐IDRI	Using Med3D models pre‐trained on ResNets, initialize classification networks using Med3D models	Accuracy	0.9192
2020	Harsono et al.^81^	LIDC‐IDRI	Integrated modified pre‐trained inflated 3D ConvNct with FPN	AUC	0.8184
2020	Yang et al.^116^	LIDC‐IDRI	Self‐attention transformer based on 3D DenseNets and MIL algorithms	AUC	0.932
2021	Yu et al.^103^	LIDC‐IDRI	Res‐trans networks	Accuracy	0.9292
				AUC	0.9628
2021	Halder et al.^117^	LIDC‐IDRI	Two‐path morphological 2D CNN	Accuracy	0.9610
				AUC	0.9936
2019	Xie et al.^78^	LIDC‐IDRI	MV‐KBC model can learn 3‐D lung nodule characteristics by decomposing a 3D nodule into nine fixed views	Accuracy	0.916
				AUC	0.957
2018	Zhu et al.^50^	LIDC‐IDRI	R‐CNN‐GBM	Accuracy	0.9274
2019	Nasrullah et al.^61^	LIDC‐IDRI	CMixNet‐GBM	Sensitivity	0.94
2023	Mikhael et al.^118^	NLST	3D Resnet	AUC	0.92
2023	Bushara et al.^119^	LIDC	LCD‐CapsNet	Accuracy	0.94
				AUC	0.989
2023	Irshad et al.^120^	Exasens dataset	An IGWO‐based DCNN model	Accuracy	98.27%
				Sensitivity	97.67%

Abbreviations: 2D, two‐dimensional; 3D, three‐dimensional; AUC, area under the curve; CNN, convolutional neural network; NLST, National Lung Screening Trial.

## 5 PREDICTION AND PROGNOSTICATION

The successful application of AI in medical diagnosis has led to increased interest in utilizing AI‐based imaging analysis to address complex clinical challenges in cancer diagnosis. Advances in computer vision and pattern recognition have enabled the development of AI‐based imaging biomarkers that are quantitative representations of tumor characteristics derived from radiological images and correlated with clinical outcomes. There are two main categories of AI‐based radiological biomarkers: Radiomics and AI. Radiomics involves manually outlining the region of interest, extracting quantitative features such as morphology, volume, intensity, texture, heterogeneity, and peritumor features, and then using an ML model to predict clinical outcomes based on these feature representations. In AI methods, a DL neural network is trained on a large dataset to learn novel representations that can be used for predictions. This chapter focuses on the predictive ability of AI for the diagnosis of early‐stage LC.

AI can predict nodule growth trends. Qi et al. studied the progression of persistent pure ground‐glass nodules (pGGNs) utilizing DL for nodule segmentation. The study analyzed 110 pGGNs from 110 patients with long‐term follow‐up using the Dr. Wise system, which utilizes a CNN to automatically segment the pGGNs from initial and subsequent CT scans. Research indicates that the growth of persistent pGGNs is most likely to follow an exponential growth model. Within the first 35 months of follow‐up, the growth rate of pGGNs remains relatively constant and then gradually slows down. It has also been found that pGGNs exhibiting lobulation and a larger initial diameter, volume, and mass are more likely to exhibit growth.^12^ Another study employing a volumetric segmentation technique to analyze the growth trends of subsolid nodules with different pathological types revealed that the exponential model (with determination coefficients of 0.89 and 0.95) better captured the overall growth and solid component growth compared to quadratic, linear, or power‐law models. Faster total volume growth was associated with a history of lung cancer, baseline nodule volume <500 mm^3^, and histopathological results indicating invasive adenocarcinoma. Non‐invasive adenocarcinoma exhibited a significantly longer median volume doubling time compared to invasive adenocarcinoma.^13^

AI can predict the histological types of LC. Guo et al. developed two automated classification models to distinguish between the different histological types and subtypes of LC (small cell lung cancer, SCLC; adenocarcinoma, ADC; squamous cell carcinoma, SCC) using non‐enhanced CT images. The first model, ProNet, is a 3D CNN that employs a ResNet‐style skip connection mechanism. Based on the test data, ProNet achieved an overall accuracy of 72% and an AUC of 84%. The second model, comradNet, is based on radiomics and comprises four fully connected layers. PyRadiomics was used to extract 1743 radiomic features, and after feature selection, 20 features were fed into ComradNet. The overall accuracy of com radNet was 75%, and its AUC was 79%. Although both models successfully differentiated SCLC, ADC, and SCC, ProNet performed better than com radNet.^82^

For the prognosis of patients with LC, Kim et al. created a CNN model to examine preoperative CT scans for predictive performance. The model was initially trained, adjusted, and validated using a dataset of patients with T1‐4N0M0 ADC. For external validation, the model was tested on a separate dataset of patients with stage I (T1‐2aN0M0) ADC. In addition, the model considers relevant clinical risk factors. Cox regression analysis was utilized to assess the impact of various factors on disease‐free survival, quantified by hazard ratios (HRs). The analysis revealed that patients with stage I lung ADC undergoing surgery can benefit from the predictions made by this DL algorithm based on their chest CT scans.^83^ Shimada et al. conducted a study to evaluate the effectiveness of using radiomics in conjunction with AI to predict early recurrence (within 2 years after surgery) in patients with clinical stage 0‐IA NSCLC. The study analyzed data from 642 patients with early recurrence who were divided into a derivation cohort and a validation cohort with a 2:1 ratio. The AI software Beta Version (Fujifilm Corporation, Japan) was used to extract 39 imaging factors from nodule characterization analysis, including 17 AI GGN analysis factors and 22 radiomic features. These results indicate that the combination of CT‐based radiomics and AI can effectively categorize the postoperative recurrence population and noninvasively predict early recurrence in patients with clinical stage 0‐IA NSCLC.^84^

A new, fully automated AI system (FAIS) that predicts the EGFR genotype was developed in the latest prospective multi‐center study published in The Lancet Digital Health. The study included 18,232 LC patients from nine cohorts in China and the United States who underwent CT scans and genetic sequencing. The FAIS achieved an AUC of 0.748–0.813 in six retrospective and prospective test cohorts, outperforming commonly used traditional tumor‐based DL models.^85^ Wang et al. created a DL model to forecast EGFR mutations in LC patients using non‐invasive CT scans. Information from 844 patients with LC at two hospitals, including preoperative CT scans (14,926 images), EGFR mutations, and patient details was analyzed. The first 20 layers of the model were trained using 1.28 million natural images from ImageNet through transfer learning. The CT images were then processed using an end‐to‐end algorithm to predict the EGFR mutation status. This model predicts the probability of an EGFR‐mutant tumor directly from a CT image without requiring additional image processing or segmentation.^86^

## 6 BIOMARKERS

The advantage of LDCT lies in its simplicity and high sensitivity, with the current definition of positive nodules primarily based on the size and/or volume of the nodules. However, to address the prevalent issue of high false‐positive rates in screening, even with refined definitions of positive nodules, new screening indicators are needed to complement and improve the existing screening systems. Thus, an evidence‐based biomarker for an overall risk assessment could be a future direction.^87^

Research findings have indicated that the application of a microRNA signature classifier (MSC) is capable of decreasing the false‐positive rate associated with LDCT by up to 80% and the sensitivity increased from 84% of LDCT alone to an impressive 98%.^88^ Serum microRNA testing has a negative predictive value greater than 99%. This implies that individuals who test negative can safely avoid subsequent LDCT follow‐ups. Studies have shown that ML models based on serum RNA levels can predict the occurrence of LC several years before diagnosis or the appearance of symptoms. The study collected 1061 samples from 925 patients within 10 years before LC diagnosis, performing an average of 18 million RNA sequencing per sample. The average AUCs for NSCLC prediction models 0–2 years and 6–8 years before diagnosis were 0.89 (95% CI, 0.84–0.96) and 0.82 (95% CI, 0.76–0.88), respectively.^89^

AI can be utilized for the detection, diagnosis, and prognosis of LC, while biomarkers are also needed to refine screening criteria for participants, aiming to reduce the costs associated with LC screening. The trends in LC screening include the integration of LDCT with biomarkers and the intersectional application of AI in molecular biology. Although there may be significant expenses in the short term, the continuous advancement of AI and the development of novel biomarkers undoubtedly present vast potential and opportunities for improvement. The long‐term outcomes are expected to be more efficient and promising.

## 7 DISCUSSION

Compared to conventional ML approaches, CNNs have shown remarkable advantages in the field of medical image analysis, particularly in various facets of lung imaging—including but not limited to lung segmentation, nodule detection, and nodule classification, as well as predictive and prognostic evaluations. As a result, CNNs have emerged as a more effective alternative for medical image analytics. In this section, we delve into the key factors that contribute to the performance gap between CNNs and traditional methodologies, along with the associated challenges and prospective directions.

### 7.1 Advantages of CNNs

The principal advantage of CNNs over conventional ML algorithms lies in their robust feature extraction capabilities. CNNs are designed to autonomously learn both high‐level and nuanced deep‐level features from image data. These features can encompass various attributes of nodules such as shape, size, density, and texture, thereby enhancing the accuracy of nodule detection. This is particularly vital for identifying intricate image characteristics that may correlate with specific pathological or genetic subtypes, as well as prognostic indicators.^82,85^ In contrast, traditional ML approaches often depend on hand‐engineered features, which may lack the depth and complexity required to capture subtle but critical information embedded within the images. Moreover, the deep architecture of CNNs enables the nonlinear and multi‐scale processing of image data. This multi‐scale perspective is crucial for understanding that lung nodules may manifest diverse characteristics at different resolutions or scales. Through the utilization of convolutional kernels and pooling layers of variable dimensions, along with techniques for multi‐scale feature fusion, CNNs are adept at conducting scale‐sensitive image analysis. Given the complex nonlinear associations that may exist among lung nodule features such as shape, size, and texture, CNNs employ nonlinear activation functions. This allows the model to capture and understand these nonlinear relationships effectively, which is crucial for accurate nodule classification.^90^, ^91^ Next, the intricate architecture of CNNs endows them with greater flexibility, allowing them to adapt to a wider array of data distributions and relational patterns. In addition to capturing the intrinsic features of nodules, CNNs can account for the contextual elements such as the adjacent tissue structure and background, factors that may be significant for pathological or genetic subtyping and prognostic evaluation. Simultaneously, CNNs are proficient at discerning the spatial relationships between lung nodules and their immediate environment. This capability is especially beneficial for the detection of ambiguous or subtle nodules that pose challenges to medical interpretation.^59,68^ Conventional methods may place excessive emphasis on local features, thereby risking the omission of vital contextual information surrounding the nodules. Moreover, CNNs utilize large datasets with extensive annotations for training to counteract the risk of overfitting. By training on such comprehensive datasets, CNNs are better equipped to generalize across a variety of lung nodule conditions. When provided with adequate data, these networks can deliver outstanding performance. Leveraging GPU acceleration, CNNs enable near‐real‐time detection of lung nodules, thus facilitating rapid responses to clinical feedback.^61^ As new data become available, CNNs can be efficiently fine‐tuned and updated, unlike traditional ML models that may require exhaustive retraining. Additionally, CNNs offer the benefit of knowledge transfer between related tasks, thereby accelerating the training phase and augmenting overall performance.^81^ These CNN architectures can also seamlessly integrate with other ML models, further enhancing the robustness of the entire system.^50,61^ Lastly, some advanced CNN architectures incorporate visualization algorithms such as gradient‐weighted class activation mapping (Grad‐CAM) and SHapley Additive exPlanations (SHAP) to tackle the “black‐box” issue often inherent in DL models. By equipping CNNs with augmented localization capabilities and integrating Shapley values from game theory, these methodologies offer not only visualization but also interpretability for the underlying decision‐making mechanisms within data‐driven DL frameworks.^92^, ^93^

### 7.2 Challenges

While the application and impact of AI in medical diagnosis have been the subjects of extensive study, its efficacy and potential are intricately linked to overcoming the challenges that presently limit its broader adoption in the field of medical imaging. These challenges, discussed below, are not only barriers to performance optimization but are also factors that can potentially impede the trust radiologists place in AI‐driven results.

#### 7.2.1 Scarcity of comprehensive and well‐annotated datasets

It is widely accepted that a substantial quantity of well‐labeled data is imperative to develop an effective DL model for medical imaging analysis. Although LC is one of the few diseases for which public datasets are available to train AI systems, there are still inconsistencies in the labeling of lung CT scan datasets, leading to variations in annotations across different datasets. Acquiring vast amounts of lung CT data with precise labels remains challenging. The collection of individual lung CT scans may be hindered by privacy concerns, and by certain hospital restrictions and national policies related to the protection of personal information. In addition, radiologists require considerable time to annotate medical images, and assigning this task to someone without the necessary competence may result in inaccurate classifications.

#### 7.2.2 Poor interpretability of diagnostic result

Using CNN‐based models, nodules can be automatically identified and classified. However, pathogenic explanations are not provided. Radiologists must be able to interpret models to determine the exact cause of the disease. Radiologists cannot make an accurate diagnosis or formulate an appropriate treatment plan based solely on detection results or diagnosis scores. Consequently, it is crucial to pay attention to CNN‐based models, which may reveal connections between input data and diagnostic results and indicate which nodule characteristics are associated with the existence of cancer.

#### 7.2.3 Challenges with the generalization ability

In the realm of medical diagnostics, a multitude of DL‐based models have been developed to tackle a broad spectrum of diagnostic challenges. While these models often exhibit remarkable performance and accuracy within their specific use‐cases, a pervasive issue remains: models that excel in one specialized task frequently struggle to generalize effectively to other, even subtly different tasks. Inferior generalization capabilities could heighten the likelihood of both misdiagnoses and missed diagnoses, posing significant risks to patient health and the efficacy of subsequent treatment strategies.

### 7.3 Future directions

First, to address the issue of dataset scarcity, data augmentation techniques, such as cropping, rotation, flipping, and proper labeling, can be employed to enhance both the quantity and diversity of datasets. Additionally, the use of generative adversarial networks can be leveraged to generate additional synthetic images and serve as a complementary source of data.^94^ It is possible to train advanced off‐the‐shelf CNNs using semi/unsupervised and self‐supervised learning methods on raw CT scans without labels when sufficient raw CT images are available, which will lead to achieving a higher level of performance than supervised learning techniques.^95^, ^96^ The accuracy of nodule identification and classification tasks with limited data can be improved through utilizing transfer learning techniques by pre‐training 3D CNNs on extensive datasets.

Second, people often focus on the performance metrics of CNN models at the expense of neglecting the interpretability of the results. Enhancing the interpretability of DL‐based models serves not only to clarify how predictions are generated, but also to gain a clear understanding of how outcomes for specific patients are obtained. This has the potential to contribute to the formulation of more accurate and reliable clinical decision‐making guidelines. Using the Markov Chain Monte Carlo technique, a BN‐based inference model was designed to enhance the interpretability of CNN‐based systems.^97^ In addition, a cause‐and‐effect inference could be extended to the task of predicting features and categorizing benign and malignant tumors. The diagnostic results can be causally correlated with the predicted feature scores.^98^

Third, employing a multi‐task learning paradigm allows the model to learn multiple related tasks simultaneously while sharing certain model parameters, thereby enhancing the model's generalization capabilities.^68^ Leveraging cloud computing technology, diagnostic records can be sent to cloud storage to update the training dataset, enabling the proposed CNN to be trained on a cloud backend to continuously adapt to real‐time changes.^70^ Given that various medical scanning devices operate in diverse settings and involve multiple imaging modalities, these factors could potentially compromise the generalizability of DL models. Therefore, a deeper exploration into how scanning parameters and image reconstruction techniques specifically affect model performance, followed by optimization tailored to these different device settings, may enhance the model's generalization capabilities.

Beyond the aforementioned future directions, assessing the efficacy of AI in the detection of solid nodule cancers with confirmed pathology is imperative instead of relying on the radiologists' consensus on suspicious nodules. Further studies evaluating the performance of innovative AI systems based on DL should be conducted using multi‐center evaluations. The influence of an AI‐generated risk score on the performance of radiologists must also be analyzed in multi‐center studies. Additionally, the possibility and feasibility of integrating AI‐generated risk scores into nodule follow‐up protocols should be considered.

### 7.4 CONCLUSION

With the advancements and implementation of cutting‐edge technologies, such as neural networks and DL algorithms, the potential for AI applications in LC screening has been continuously explored. AI plays a crucial role in lung segmentation, nodule detection, false‐positive reduction, nodule classification, prediction, and prognosis assessment. AI offers an objective, efficient, multivariate, and reproducible approach to these tasks, thereby reducing the burden on clinicians, minimizing misdiagnoses due to fatigue, and potentially transforming current medical models.

AI models are increasingly applied to various data sources, including clinical information, imaging histology, histopathology, and molecular biomarkers, to improve the accuracy of assessment of disease risk, detection, and treatment response prediction. Despite these promising results, AI is still in its early stages and has limitations when applied to LC screening, thus requiring further exploration and improvement to standardize AI data and enhance the generalizability and interpretability of the results. Future research should focus on the large‐scale validation of novel algorithms based on DL and the initiation of multi‐center clinical studies to verify the effectiveness of CNN‐based automated categorization in improving patient outcomes. The integration of AI algorithms can assist well‐trained readers in classifying normal scans and has the potential to improve screening cost‐effectiveness. Although further research is warranted, it is clear that AI will play a leading role in LC screening in the coming decades.

## 8 APPENDIX

In this benchmark analysis, we followed a four‐step methodology: (1) keywords were searched in multiple academic databases (IEEE Xplore, Scopus, Google Scholar, Science Direct, PubMed, and Web of Science); (2) relevant studies were collected and duplicates were removed; (3) selection criteria were applied to focus on AI technologies using CT images for lung cancer screening, including lung segmentation, nodule detection, nodule classification, benign‐malignant nodule analysis, and nodule prognosis; and (4) system performance was evaluated using established metrics. For our search, we employed an array of keywords including “lung cancer,” “pulmonary nodule,” “lung nodule,” “segmentation,” “detection,” “classification,” “false positive reduction,” “prediction,” “prognosis,” “CNN,” “convolutional neural network,” “deep learning,” “artificial intelligence,” and “AI.” These keywords were strategically combined using the Boolean operators “OR” and “AND” to optimize the comprehensiveness and specificity of our search results.

## AUTHOR CONTRIBUTIONS

**Wu Quanyang:** Conceptualization (equal); data curation (equal); investigation (equal); visualization (equal); writing – original draft (equal). **Huang Yao:** Resources (equal); supervision (equal). **Wang Sicong:** Formal analysis (equal); supervision (equal). **Qi Linlin:** Supervision (equal); visualization (equal). **Zhang Zewei:** Data curation (equal); investigation (equal). **Hou Donghui:** Investigation (equal). **Li Hongjia:** Investigation (equal). **Zhao Shijun:** Funding acquisition (equal); project administration (equal); supervision (equal); writing – review and editing (equal).

## FUNDING INFORMATION

This work was supported by the National Key R&D Program of China (2020AAA0109504) and the CAMS Innovation Fund for Medical Sciences (2021‐I2M‐C&T‐B‐063).

## CONFLICT OF INTEREST STATEMENT

The authors declare that the research was conducted in the absence of any commercial or financial relationships that could be construed as a potential conflict of interest.

## Supporting information


Data S1:

## Data Availability

The datasets used in this study can be obtained from the corresponding author upon a reasonable request.
