# Supplementary material for: Artificial intelligence in lung cancer screening: Detection, classification, prediction, and prognosis
Source: Cancer Med. 2024 Apr 5;13(7):e7140. doi: 10.1002/cam4.7140 (PMC10997848; doi:10.1002/cam4.7140)
Supplement: Supplementary file 1 — Data S1: [file CAM4-13-e7140-s001.pdf]

Supplementary Material

we have provided descriptions of the mechanisms of 12 different CNN models, along with their corresponding references.

1. LeNet: The LeNet architecture is very compact and simplified. LeNet was proposed by Yann LeCun in 1989.(1) There are several versions of LeNet, including LeNet-5, LeNet-4, LeNet-1, and Boosted LeNet-4. LeNet is used for handwritten character recognition. LeNet is a classic convolutional neural network that employs convolution, pooling, and fully connected layers. It has been utilized for the handwritten digit recognition task on the MNIST dataset (a dataset of 60,000 grayscale images of single digits between 0 and 9, each in a 28 × 28 pixel square).

2. AlexNet: AlexNet was introduced by Alex Krizhevsky.(2) AlexNet is a pioneering convolutional neural network (CNN) architecture that played a crucial role in advancing the field of deep learning, particularly in image classification tasks.

Key Components and Innovations:

Deep Architecture: AlexNet was one of the first CNNs to exhibit a deep architecture, consisting of multiple convolutional and fully connected layers. It comprised eight layers: five convolutional layers followed by three fully connected layers.

Convolutional Layers: Convolutional layers are responsible for learning hierarchical features from input images. AlexNet employed rectified linear units (ReLUs) as activation functions, enhancing the network's ability to model complex nonlinear relationships.

Local Response Normalization (LRN): A novel innovation in AlexNet was the incorporation of LRN layers after certain convolutional layers. LRN aimed to enhance the network's generalization by normalizing responses within local neighborhoods and facilitating competition between adjacent neurons.

Overlapping Pooling: Traditional pooling layers were replaced with overlapping max-pooling layers, reducing overfitting while preserving spatial information.

Data Augmentation: To tackle limited training data, AlexNet used data augmentation techniques such as image translations, reflections, and color alterations. This improved the network's robustness to variations in input images.

Dropout Regularization: Dropout, a regularization technique, was introduced in the fully connected layers. Dropout randomly deactivates a fraction of neurons during training, preventing the network from relying heavily on specific neurons and promoting generalization.

Large-Scale Training: AlexNet was trained on the massive ImageNet dataset, comprising millions of labeled images across thousands of classes. To facilitate this, the authors employed GPUs, which significantly accelerated training times.

AlexNet's groundbreaking achievements inspired the development of more advanced architectures like VGG, GoogLeNet, and ResNet. The principles introduced by AlexNet laid the foundation for the deep learning revolution, impacting various domains beyond computer vision, including natural language processing and reinforcement learning.

3. VGG: The VGG (Visual Geometry Group) framework was devised by A. Zisserman and K. Simonyan at the University of Oxford .(3)The VGGNet architecture comprises 16 layers, consisting of 13 convolutional layers, 5 pooling layers, and 3 fully connected layers.

Key Components and Innovations

Uniform Architecture: VGG's design principle revolves around using a uniform architecture with a consistent

configuration of convolutional and pooling layers. This architecture is characterized by its deep stack of simple, small-sized convolutional filters.

Convolutional Layers: VGG employs a series of convolutional layers with 3x3 filters. These smaller filters are stacked multiple times to learn intricate patterns and features at various scales.

Pooling Layers: VGG uses max-pooling layers with a fixed 2x2 filter size and a stride of 2. This operation reduces spatial dimensions while preserving essential features.

Deep Stacking: One of VGG's notable characteristics is its depth, with models ranging from VGG11 to VGG19. Deeper networks allow the model to learn complex hierarchical features, improving its ability to discriminate between different classes.

Use of Fully Connected Layers: VGG typically concludes with a few fully connected layers. These layers process the high-level features extracted by the preceding convolutional and pooling layers to make class predictions.

Activation Functions: VGG primarily uses rectified linear units (ReLUs) as activation functions after each convolutional and fully connected layer. ReLUs address the vanishing gradient problem and accelerate convergence.

While VGG's design is less streamlined compared to later architectures like GoogLeNet and ResNet, its impact on the field of deep learning cannot be understated. It provided valuable insights into the role of depth in learning and paved the way for more sophisticated and efficient CNN designs that followed.

4. GoogleNet: In 2014, a team at Google introduced the GoogleNet architecture. It employs a convolutional neural network inspired by the LeNet CNN and incorporates the innovative Inception module. GoogleNet also utilizes techniques such as RMSprop, image distortions, and batch normalization.(4)

The GoogleNet architecture encompasses 22 layers, incorporating a total of 9 inception modules.

The initial layer serves as the input layer, accommodating a 224 × 224 × 3 RGB image. Initially, a convolutional technique is applied using a filter size of 7 × 7 and a stride of 2. This leads to a layer size of 112 × 112 × 64, with 64 representing the number of used filters.

Subsequently, the pooling technique is implemented through max pooling with a filter size of 3 × 3 and a stride of 2. This results in a layer size of 56 × 56 × 64, featuring 64 utilized filters.

Convolution is then applied with a 3 × 3 filter and a stride of 1, resulting in a layer size of 56 × 56 × 192, with 192 filters employed.

Recurring application of max pooling with a filter size of 3 × 3 and a stride of 2 yields a layer size of 28 × 28 × 192, utilizing 192 filters.

The inception technique is introduced, involving 3 filters (1 × 1), (3 × 3), (5 × 5), followed by max pooling. This leads to a subsequent layer size of 28 × 28 × 256.

Continuing this pattern, the inception technique is repeatedly applied, resulting in a layer size of 28 × 28 × 480.

Max pooling is applied with a filter size of 3 × 3 and a stride of 2, leading to a layer size of 14 × 14 × 480, with 480 filters in use.

The inception technique is employed 5 times, yielding a next layer size of 14 × 14 × 832.

Further, max pooling is applied with a filter size of 3 × 3 and a stride of 2, resulting in a layer size of 7 × 7 × 832, with 832 filters utilized.

The inception process is repeated twice, resulting in a layer size of 7 × 7 × 1024.

Consistently, average pooling is applied with a filter size of 7 × 7 and a stride of 1. This leads to a subsequent layer size of 1 × 1 × 1024, and after applying a 40% dropout, the final output becomes 1 × 1 × 100.

5. ResNet: ReNet is a neural network architecture designed for processing image data.(5) It represents a variant

of recurrent neural networks (RNNs).

The core idea of ReNet involves employing recurrent neural networks in various directions across an image to capture spatial relationships and structural information. While traditional convolutional neural networks (CNNs) use fixed-size convolutional kernels to extract features from images, ReNet applies RNNs to each image block.

The operational process of ReNet can be outlined as follows:

Divide the input image into distinct blocks, such as employing a grid-based partitioning.

Within each block, utilize RNNs to process sequences of pixels. RNNs can be basic LSTM (Long Short-Term Memory) or GRU (Gated Recurrent Unit) models.

ReNet runs RNNs in different directions (horizontal, vertical, diagonal, etc.), capturing features and relationships in various image directions.

Finally, combine the outputs of RNNs from each direction to obtain a holistic representation of image features.

ReNet's strength lies in its effective capture of spatial relationships within images, especially when dealing with temporal sequence image data like videos. However, due to its relatively higher computational complexity, it might encounter challenges when processing large-sized images.

These are the functions of a Residual Network.

$y = F(x, W_i) + x$

$y = F(x, W_i) + W_{sx}$

These two are the equations used where x & y are input and output vectors.

6. DenseNet: DenseNet is utilized for object recognition and has demonstrated superior performance compared to ResNet in this domain. While the architectures of DenseNet and ResNet share similarities, DenseNet's slight modification is pivotal in achieving superior outcomes over ResNet. Notably, DenseNet employs layer concatenation, whereas ResNet employs an additive approach. It's worth mentioning that DenseNet's utilization of concatenation necessitates GPU support.(6)

The DenseNet is a neural network architecture designed to address the vanishing gradient problem and enhance information flow in deep networks.

The core principle of DenseNet involves dense connections between layers, where each layer receives feature maps from all preceding layers, promoting feature reuse and strengthening gradient flow. This concept stands in contrast to traditional architectures where layers are connected sequentially. DenseNet is particularly effective in alleviating vanishing gradient issues and improving the flow of gradients during training, leading to enhanced convergence and information propagation.

The main characteristics of DenseNet are:

Dense Blocks: In a DenseNet, the network is organized into dense blocks, each consisting of multiple convolutional layers. Within a dense block, each layer receives the feature maps from all preceding layers, including its own feature maps. This dense connectivity fosters rich feature representations and encourages feature reuse.

Transition Layers: Between dense blocks, transition layers are introduced to control the growth of the network and reduce computational complexity. Transition layers include convolutional and pooling operations to reduce the spatial dimensions of feature maps.

Bottleneck Layers: Within each dense block, bottleneck layers may be employed to reduce the number of input feature maps before passing them on to subsequent layers. This helps manage computational costs.

The benefits of DenseNet include efficient parameter usage, as well as enhanced information flow and gradient propagation due to the dense connections. The architecture also mitigates the risk of overfitting and exhibits strong performance even with limited training data. DenseNet has shown remarkable results on various computer vision

tasks, including image classification, object detection, and segmentation.

7. U-Net: U-Net is a convolutional neural network that was introduced for image segmentation in the biomedical field. It was introduced by Olag Ronneberger in 2015.(7)

The core principle of the U-Net revolves around a U-shaped architecture with an encoder-decoder structure, featuring skip connections to preserve spatial information and facilitate accurate segmentation. This design is particularly effective in scenarios where precise localization of objects in images is essential.

The main components of the U-Net architecture are:

Encoder: The encoder portion of the U-Net consists of a series of convolutional and pooling layers that progressively reduce the spatial dimensions of the input image. This helps extract high-level features and semantic information from the input.

Decoder: The decoder portion of the U-Net aims to upsample the feature maps to the original input resolution. It comprises a sequence of upsampling and convolutional layers. Each upsampling step expands the feature maps to restore spatial information.

Skip Connections: One of the distinctive features of U-Net is the incorporation of skip connections. These connections connect corresponding encoder and decoder layers, allowing the decoder to access high-resolution features from the encoder. Skip connections facilitate accurate localization by enabling the network to combine detailed local information from the encoder with contextual information from the decoder.

Final Layer: The final layer typically employs a convolutional layer with a softmax activation to generate pixel-wise segmentation probabilities. Each pixel is assigned a class label indicating the presence or absence of the target object.

U-Net's architecture is well-suited for applications where pixel-level segmentation accuracy is crucial, such as medical image segmentation, cell segmentation, and similar tasks. The skip connections contribute to its ability to capture fine details while maintaining a broader context. U-Net has demonstrated impressive performance in various segmentation challenges and remains a popular choice in the field of computer vision.

8. V-Net: The V-Net is a three-dimensional convolutional neural network architecture primarily designed for volumetric medical image segmentation tasks. It was introduced by Milletari.(8)

The core principle of the V-Net centers on extending the concept of U-Net to 3D volumes, making it suitable for segmenting volumetric medical data such as MRI or CT scans. The architecture's design aims to accurately delineate structures within three-dimensional images, which is crucial in medical image analysis.

The main components and operation of the V-Net architecture include:

Encoder-Decoder Structure: Similar to the U-Net, the V-Net follows an encoder-decoder structure. The encoder extracts high-level features through convolutional and pooling layers, while the decoder upsamples and generates segmentations.

3D Convolution and Deconvolution: Given that medical images are volumetric, the V-Net employs 3D convolutions for feature extraction and 3D deconvolutions for upsampling. This enables the network to capture spatial relationships and structural information within the volume.

Residual Connections: The V-Net incorporates residual connections between corresponding encoder and decoder layers. These connections aid in gradient propagation and assist in alleviating the vanishing gradient problem, contributing to more stable and efficient training.

Volumetric Segmentation: The final layer of the V-Net comprises a 3D convolutional layer followed by a softmax activation function. This generates volumetric segmentations with class probabilities assigned to each voxel, indicating the presence or absence of structures of interest.

The V-Net architecture's suitability for volumetric medical image segmentation tasks arises from its ability to process 3D volumes while preserving spatial information and contextual details. The incorporation of residual connections enhances its training efficiency and convergence.

9. SegNet: SegNet, developed by researchers at the University of Cambridge, is a convolutional network designed specifically for multiclass pixel-wise segmentation tasks.(9) The architecture of SegNet comprises an encoder and a decoder.

In the encoder stage, convolutional layers are utilized along with batch normalization and ReLU activation, followed by non-overlapping max pooling to achieve downsampling. This process involves a total of 13 convolutional layers adapted from the VGG-16 model. Importantly, the locations of max pooling are stored to facilitate subsequent upsampling.

The decoder phase involves convolutional operations as well as upsampling. To accomplish this, the max pooling indices from the encoder stage are used to guide the upsampling process, effectively reconstructing the spatial dimensions. At the conclusion of the decoder, a softmax classifier is applied to each pixel, enabling the prediction of class labels.

In the visual representation provided, the relationship between upsampling and the corresponding encoder layer is illustrated. This process, referred to as "up sampling," contributes to the accurate restoration of spatial information. Ultimately, a softmax classifier at the end of the network assigns class predictions to individual pixels.

10. Fast-RCNN: Fast R-CNN is a neural network architecture designed for efficient object detection in images. It improves upon the previous R-CNN model by introducing several innovations to streamline the detection process. (10)

The key principle of Fast R-CNN is to perform object detection and classification using a single, unified network. It combines region proposal generation, feature extraction, and object classification into a single forward pass, eliminating the need for separate steps as in R-CNN.

The main components and working mechanism of the Fast R-CNN architecture are as follows:

Region Proposal Network (RPN): Instead of relying on external methods like Selective Search for region proposals, Fast R-CNN employs an internal RPN that generates region proposals directly from the input image. The RPN operates on shared convolutional features and predicts objectness scores and bounding box offsets for potential regions.

RoI Pooling: After generating region proposals, RoI (Region of Interest) pooling is applied to align the regions with fixed-size feature maps that can be fed into a neural network. This allows the extraction of consistent features for each region, regardless of its size or location.

Feature Extraction: The aligned RoIs are passed through a series of fully connected and convolutional layers to extract relevant features. This process yields a fixed-length feature vector for each RoI.

Object Classification and Localization: The extracted features are used for object classification and bounding box regression. Class probabilities are predicted for each RoI, along with refined bounding box coordinates.

The main advantage of Fast R-CNN is its efficiency. By sharing convolutional features across the entire image and RoIs, it significantly reduces computational redundancy compared to the sequential processing of R-CNN. This results in faster inference times without compromising detection accuracy.

Fast R-CNN's streamlined approach to object detection makes it an effective choice for real-time applications where speed and accuracy are crucial. It combines object proposal generation, feature extraction, and object classification into a single framework, leading to more efficient and accurate object detection compared to its predecessors.

11. Mask R-CNN: Mask R-CNN is a designed for accurate instance segmentation, which involves both object detection and pixel-wise object mask prediction. It extends the Fast R-CNN architecture by incorporating an additional branch to predict object masks along with class labels and bounding box coordinates. Mask R-CNN was introduced by Kaiming He et al. in the paper "Mask R-CNN" . (11)

The central principle of Mask R-CNN is to enhance object detection with precise pixel-level segmentation. It achieves this by simultaneously predicting object classes, bounding box coordinates, and object masks for each instance in an image.

The key components and working mechanism of the Mask R-CNN architecture are as follows:

Region Proposal Network (RPN): Similar to Fast R-CNN, Mask R-CNN uses an internal RPN to generate region proposals directly from the input image. These proposals serve as potential regions of interest.

RoI Align: After generating region proposals, RoI Align is employed to obtain fixed-size feature maps for each region. RoI Align overcomes the misalignment issues associated with RoI Pooling, ensuring accurate feature extraction for mask prediction.

Feature Extraction: Extracted RoI features are processed through a series of convolutional and fully connected layers, leading to class predictions and refined bounding box coordinates as in Fast R-CNN.

Mask Prediction: A parallel mask branch is added to the network, consisting of additional convolutional layers. This branch generates binary masks that correspond to the pixel-wise segmentation of each object instance.

Bounding Box Regression and Mask Refinement: The predicted masks are refined using the bounding box information, aligning the masks with the object's spatial extent.

Mask R-CNN's notable advantage lies in its ability to provide accurate object segmentation along with detection. By introducing the mask branch, it facilitates instance-level segmentation, where each object instance is accurately delineated at the pixel level.

Mask R-CNN is widely used in applications requiring detailed object segmentation, such as medical image analysis, scene understanding, and robotics. Its integration of object detection and pixel-wise mask prediction makes it a powerful tool for tasks demanding both object localization and precise segmentation.

12. RetinaNet: RetinaNet is a single-stage object detection model proposed by Lin et al.(12) It addresses the challenge of detecting objects at different scales and levels of detail in an image. RetinaNet combines a one-stage architecture with a novel loss function called "Focal Loss" to achieve accurate and efficient object detection.

Here's a brief explanation of the RetinaNet principles:

Feature Pyramid Network (FPN) Backbone: RetinaNet employs a Feature Pyramid Network (FPN) as its backbone architecture. FPN generates a multi-scale feature pyramid by combining features from different levels of a pre-trained convolutional neural network (CNN), such as ResNet. The feature pyramid allows the model to detect objects of various sizes by utilizing both high-resolution and low-resolution feature maps.

Anchor Boxes: RetinaNet uses anchor boxes to predict object locations and categories. These anchor boxes are pre-defined bounding boxes of different scales and aspect ratios, distributed across the feature map. The model predicts whether each anchor box contains an object or not and also predicts the precise bounding box coordinates and object class probabilities.

Classification and Regression Heads: RetinaNet has two parallel subnetworks attached to each level of the FPN feature pyramid: one for classifying objects and another for regressing the bounding box coordinates of the objects. The classification head predicts the probability of an anchor box containing an object for each class. The regression head refines the coordinates of the predicted bounding box based on the anchor box.

Focal Loss: The novel contribution of RetinaNet is the introduction of the Focal Loss, which addresses the issue of

class imbalance between object and background samples. Focal Loss assigns higher weights to hard-to-classify examples, i.e., the ones that are more likely to be misclassified. This helps the model focus on challenging cases during training and reduces the dominance of easy negative samples.

Loss Function: The total loss for RetinaNet is a combination of the classification loss (using Focal Loss) and the regression loss (typically using smooth L1 loss). The classification loss encourages the model to focus on hard examples, while the regression loss refines the predicted bounding boxes.

Inference: During inference, RetinaNet generates anchor box predictions across different scales and levels of the feature pyramid. It then applies non-maximum suppression (NMS) to eliminate redundant and overlapping predictions, producing the final set of detected objects along with their corresponding bounding boxes and class labels.

In summary, RetinaNet combines a feature pyramid architecture with anchor boxes and a novel Focal Loss to address the challenges of multi-scale object detection and class imbalance. This approach enables accurate and efficient object detection in various scenarios.

References

1. LeCun Y, Bottou L, Bengio Y, et al. Gradient-based learning applied to document recognition[J]. Proceedings of the IEEE, 1998, 86(11): 2278-2324. doi: 10.1109/5.726791

2. Krizhevsky A, Sutskever I, Hinton G E. ImageNet classification with deep convolutional neural networks[J]. Communications of the ACM, 2017, 60(6): 84-90. doi:10.1145/3065386

3. Simonyan K, Zisserman A. Very deep convolutional networks for large-scale image recognition[J]. arXiv preprint arXiv:1409.1556, 2014. doi:10.48550/arXiv.1409.1556.

4. J. Deng, W. Dong, R. Socher, L. -J. Li, Kai Li and Li Fei-Fei. ImageNet: A large-scale hierarchical image database. IEEE Conference on Computer Vision and Pattern Recognition, Miami, FL, USA, 2009, pp. 248-255, doi: 10.1109/CVPR.2009.5206848.

5. He K, Zhang X, Ren S, Sun J. Deep residual learning for image recognition. In: Proceedings of the IEEE conference on computer vision and pattern recognition, 2016, pp 770-778.

6. Huang G, Liu Z, Van Der Maaten L, Weinberger KQ. Densely connected convolutional networks. In: Proceedings of the IEEE conference on computer vision and pattern recognition, 2017, pp 4700–4708.

7. Ronneberger O, Fischer P, Brox T. U-net: convolutional networks for biomedical image segmentation. In: International conference on medical image computing and computer-assisted intervention, 2015, pp 234–241.

8. Ronneberger O, Fischer P, Brox T. U-net: Convolutional networks for biomedical image segmentation[C]//Medical Image Computing and Computer-Assisted Intervention–MICCAI 2015: 18th International Conference, Munich, Germany, October 5-9, 2015, Proceedings, Part III 18. Springer International Publishing, 2015: 234-241.

9. Badrinarayanan V, Kendall A, Cipolla R. Segnet: a deep convolutional encoder-decoder architecture for image

segmentation. IEEE Trans Pattern Anal Mach Intell. 2017;39(12):2481–2495. doi: 10.1109/TPAMI.2016.2644615.

10. Girshick R. Fast r-cnn. Proceedings of the IEEE international conference on computer vision. 2015: 1440-1448.

11. He K, Gkioxari G, Dollár P, et al. Mask r-cnn. Proceedings of the IEEE international conference on computer vision. 2017: 2961-2969.

12. Lin T Y, Goyal P, Girshick R, et al. Focal loss for dense object detection. Proceedings of the IEEE international conference on computer vision. 2017: 2980-2988.
